# Direct quantum process tomography via measuring sequential weak values of incompatible observables

**DOI:** 10.1038/s41467-017-02511-2

**Published:** 2018-01-15

**Authors:** Yosep Kim, Yong-Su Kim, Sang-Yun Lee, Sang-Wook Han, Sung Moon, Yoon-Ho Kim, Young-Wook Cho

**Affiliations:** 1 Department of Physics, Pohang University of Science and Technology (POSTECH), Pohang, 37673 Korea; 2 Center for Quantum Information, Korea Institute of Science and Technology (KIST), Seoul, 02792 Korea

## Abstract

The weak value concept has enabled fundamental studies of quantum measurement and, recently, found potential applications in quantum and classical metrology. However, most weak value experiments reported to date do not require quantum mechanical descriptions, as they only exploit the classical wave nature of the physical systems. In this work, we demonstrate measurement of the sequential weak value of two incompatible observables by making use of two-photon quantum interference so that the results can only be explained quantum physically. We then demonstrate that the sequential weak value measurement can be used to perform direct quantum process tomography of a qubit channel. Our work not only demonstrates the quantum nature of weak values but also presents potential new applications of weak values in analyzing quantum channels and operations.

## Abstract (web summary)

Weak measurements constitute a powerful tool for metrology, tomography and even foundations, but in most cases could be explained classically. Here, the authors characterise a quantum channel using sequential weak value measurements of two incompatible observables utilising two-photon quantum interference.

## Introduction

Measurement plays a quintessential role in quantum theory and is tied to many peculiarities of quantum physics. The projection postulate stipulates that a quantum system is irrecoverably collapsed into one of the eigenstates of the observable, resulting in maximum state disturbance. Weak measurement, however, relaxes this constraint and has enabled novel research into important problems in quantum physics and quantum information, e.g., minimum-disturbance measurement^1,2^, relation between information gain and reversibility of measurement^3^, protecting quantum states from decoherence^4,5^, measurement based quantum state manipulation^6–8^, etc. In particular, the “weak value”, obtained from a weak measurement followed by a strong measurement, is quite peculiar in that it is in general a complex number and is not bounded by the eigenvalue spectrum of the associated observable^9,10^. Since the first demonstration of the weak value concept^11,12^, the physical properties of the weak value have been studied extensively both theoretically and experimentally^13–16^ and these properties have been used to explore a variety of fundamental problems in quantum physics^17–21^. In recent years, the weak value concept is being actively exploited for quantum technology, for instance, in precision measurement and metrology^22–25^, and in quantum information as a tool for direct characterization of quantum states^26–34^.

As the weak measurement does not irrecoverably collapse the quantum state, it opens up the possibility of sequential quantum measurement for two non-commuting observables. While such sequential weak value measurement has been reported very recently^33,34^, these experiments are classical in that the results can be fully explained with the classical electromagnetic theory. Note that, despite a nonclassical single-photon state is used in ref.^34^, the weak value measuring apparatus itself is classical. In fact, it is worth noting that optical weak value experiments to date are mostly classical with a few rare exceptions^14,20^.

In this work, we report measurement of the sequential weak value of two incompatible observables by making use of two-photon quantum interference^35^ so that the results can only be explained quantum physically. We then demonstrate that the sequential weak value measurement can be used to perform direct quantum process tomography of a quantum channel. The genuine quantum nature of the sequential weak value for two incompatible observables reported in this work will be instrumental in rigorous tests quantum contextuality^36,37^ and macroscopic realism^38,39^. Moreover, direct quantum process tomography (d-QPT) based on sequential weak value measurement offers a way for characterizing quantum channels and gates. We also compare and identify advantages and disadvantages, via experiment and numerical simulation, of d-QPT via sequential weak value, standard QPT, and compressive-sensing QPT^40,41^.

## Results

### Schematic and theory

**Fig. 1:**
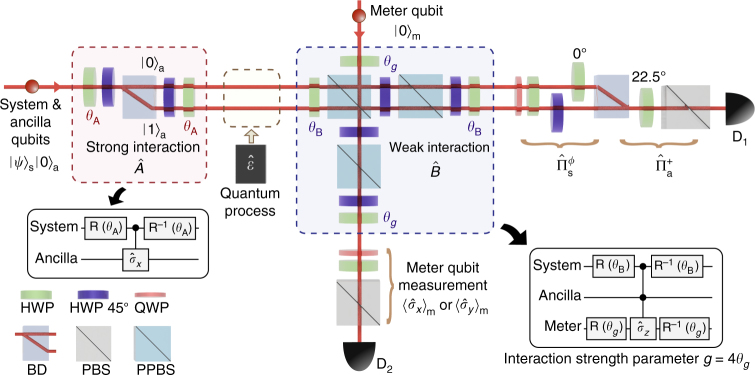
Schematic of experimental setup. The system and meter qubits are encoded in the polarization state of single-photons. The ancilla qubit is encoded on the path mode of the single-photon carrying the system qubit. Measurement of the observables $\hat A$ and $\hat B$ are sequentially applied to the system qubit |*ψ*〉_s_. Projection measurement of the observable $\hat A$, arbitrarily set by the angle *θ*_A_ of HWP, is accomplished by interacting it with the ancilla qubit |0〉_a_. Likewise, weak measurement of the observable $\hat B$, arbitrarily set by the angle *θ*_B_ of HWP, is accomplished by interacting the system and ancilla qubits with the meter qubit |0〉_m_. The weak measurement strength is parameterized by *g* = 4*θ*_*g*_ where *θ*_*g*_ is the angle of HWP. The sequential weak value $\langle {\hat B\hat A} \rangle _{\mathrm{w}}$ is obtained from the expectation values of the meter qubit conditioned on the post-selective projection measurements on the system and ancilla qubits ${{\hat {\mathrm \Pi}}}_{\mathrm{s}}^\phi$ and ${{\hat {\mathrm \Pi}}}_{\mathrm{a}}^ +$. To perform direct quantum process tomography of a quantum channel $\hat {\cal E}$ with sequential weak values, an arbitrary quantum operation $\hat {\cal E}$ is inserted between the observables $\hat A$ and $\hat B$. BD (beam displacer), PBS (polarizing beam splitter), PPBS (partially polarizing beam splitter), QWP (quarter wave plate), SPCM (single-photon counting module)

The experimental schematic of the sequential weak value measurement apparatus for two non-commuting observables $\hat A$ and $\hat B$ is shown in Fig. 1. First, two single-photons are prepared by spontaneous parametric down-conversion (SPDC). The system and ancilla qubits are encoded, respectively, on the polarization and path modes of one of the photons^42^. The meter qubit is encoded on the polarization mode of the other photon. The system qubit is measured with the help of the ancilla qubit and the meter qubit is used for the read-out of the measurement outcome. The computational basis {|0〉, |1〉} is used throughout the text with the understanding that, for the polarization qubit, it refers to horizontal and vertical polarization states and, for the path qubit, it refers to upper and lower path modes. The system qubit is prepared in an arbitrary initial state |*ψ*〉_s_ = *α*|0〉_s_ + *β*|1〉_s_ and the ancilla and meter qubits are initialized to |0〉_a_ and |0〉_m_, respectively. The three qubits are initially in the product state |*ψ*〉_s_ |0〉_a_ |0〉_m_.

To measure the sequential weak value of two non-commuting observables, measurement of the observables $\hat A$ and $\hat B$ are sequentially applied to the system qubit |*ψ*〉_s_. Measurement of the observable $\hat A$ is accomplished by interacting it with the ancilla qubit |0〉_a_. As shown in Fig. 1, the interaction $\hat U_{\mathrm{{A}}}$ between the system qubit and the ancilla qubit is a controlled-*σ*_*x*_ type operation which can be written as

1 $$\hat U_{\mathrm{{A}}} = \left( {\hat {\Bbb I} - \hat A} \right) \otimes \hat {\Bbb I} + \hat A \otimes \hat \sigma _x,$$

where $\hat {\Bbb I}$ is the identity operation. This interaction is implemented in experiment with a beam displacer (BD) and wave plates^42^. The observable $\hat A$ may be arbitrarily set by choosing the angle *θ*_A_ of HWP in Fig. 1. For instance, choosing $\theta _{\mathrm{A}} = \frac{\pi }{4},0, - \frac{\pi }{8}$, and $\frac{\pi }{8}$ rotates the bases |0〉, |1〉, |+〉, and |−〉 into |1〉, thereby implementing the observable $\hat A = \left| 0 \right\rangle \left\langle 0 \right|,\left| 1 \right\rangle \left\langle 1 \right|,\left| + \right\rangle \left\langle + \right|$, and, $\left| - \right\rangle \left\langle - \right|$ respectively. Here, $\left| + \right\rangle \equiv \left( {\left| 0 \right\rangle + \left| 1 \right\rangle } \right){\mathrm{/}}\sqrt 2$ and $\left| - \right\rangle \equiv \left( {\left| 0 \right\rangle - \left| 1 \right\rangle } \right){\mathrm{/}}\sqrt 2$.

The complete state of the three qubits after the first interaction is given by

2 $$\left( {\hat {\Bbb I} - \hat A} \right)\left| \psi \right\rangle _{\mathrm{s}}\left| 0 \right\rangle _{\mathrm{a}}\left| 0 \right\rangle _{\mathrm{m}} + \hat A\left| \psi \right\rangle _{\mathrm{s}}\left| 1 \right\rangle _{\mathrm{a}}\left| 0 \right\rangle _{\mathrm{m}}.$$

Note the measurement interaction for $\hat A$ is considered to be strong or projective in the sense that $\hat A\left| \psi \right\rangle _{\mathrm{s}}$ is completely discriminated from its orthogonal counterpart $\left( {\hat {\Bbb I} - \hat A} \right)\left| \psi \right\rangle _{\mathrm{s}}$ by the ancillary qubit state.

Measurement of the observable $\hat B$ is accomplished by interacting the system and ancilla qubits with the meter qubit |0〉_m_ as shown in Fig. 1. The interaction $\hat U_{\mathrm{B}}$ involving the three qubits is effectively a controlled–controlled-*σ*_*z*_ type operation which can be written as

3 $$\begin{array}{*{20}{l}} {\hat U_{\mathrm{B}}} \hfill & = \hfill & {\left( {\hat {\Bbb I} - \hat B} \right) \otimes \left| 1 \right\rangle \left\langle 1 \right| \otimes \hat {\Bbb I} + \hat {\Bbb I} \otimes \left| 0 \right\rangle \left\langle 0 \right| \otimes \hat {\Bbb I}} \hfill \\ {} \hfill & {} \hfill & { + \hat B \otimes \left| 1 \right\rangle \left\langle 1 \right| \otimes R^{ - 1}\left( {\theta _g} \right)\hat \sigma _zR\left( {\theta _g} \right),} \hfill \end{array}$$

where the single-qubit operation *R*(*θ*) is defined by $R\left( \theta \right)\left| 0 \right\rangle \to {\mathrm{cos}}{\kern 1pt} 2\theta \left| 0 \right\rangle + {\mathrm{sin}}{\kern 1pt} 2\theta \left| 1 \right\rangle$ and $R\left( \theta \right)\left| 1 \right\rangle \to {\mathrm{sin}}{\kern 1pt} 2\theta \left| 0 \right\rangle - {\mathrm{cos}}{\kern 1pt} 2\theta \left| 1 \right\rangle$. The rotation of angle *θ*_*g*_ determines the measurement strength *g* = 4*θ*_*g*_. For example, $\hat U_{\mathrm{B}}$ becomes the identity operation (i.e., null measurement) when *g* = 0 and, at *g* = *π*/2, it becomes the controlled–controlled–*σ*_*x*_ operation, a strong measurement. Note that the weak measurement condition is valid when $\left| g \right| \ll 1$.

In experiment, the measurement interaction for the observable $\hat B$ is realized by the two-photon quantum interference at a partially polarizing beam splitter (PPBS), where $\hat B$ is set arbitrarily by the angle *θ*_B_ of HWPs. The PPBS partially reflects vertical polarization with transmittance *T*_V_ = 1/3 and transmits horizontal polarization with *T*_H_ = 1. Since two-photon quantum interference occurs only for the ancilla qubit in the |1〉_a_ state^35^, the *π*-phase shift due to the quantum interference is only induced when the system, ancilla, and meter qubits are in the |1〉_s_ |1〉_a_ |1〉_m_. Two additional PPBS-HWP sets are used to equalize the probability amplitudes at the output ports for arbitrary input polarization states^43^.

The three-qubit quantum state after the $\hat U_{\mathrm{B}}$ interaction, evaluated up to the first-order of the weak interaction strength *g* is given by

4 $$\left| \psi \right\rangle _{\mathrm{s}}\left| 0 \right\rangle _{\mathrm{a}}\left| 0 \right\rangle _{\mathrm{m}} - \sqrt 2 \hat A\left| \psi \right\rangle _{\mathrm{s}}\left| - \right\rangle _{\mathrm{a}}\left| 0 \right\rangle _{\mathrm{m}} + g\hat B\hat A\left| \psi \right\rangle _{\mathrm{s}}\left| 1 \right\rangle _{\mathrm{a}}\left| 1 \right\rangle _{\mathrm{m}},$$

where the last term clearly shows that the result of the sequential observables $\hat B\hat A$ is registered on the meter. The superfluous second term can be eliminated by subjecting the ancilla qubit into projection measurement ${{\hat {\mathrm \Pi}}}_{\mathrm{a}}^ + = \left| + \right\rangle \left\langle + \right|$^44^, resulting in the joint state of the system and the meter qubits

5 $$\left| \psi \right\rangle _{\mathrm{s}}\left| 0 \right\rangle _{\mathrm{m}} + g\hat B\hat A\left| \psi \right\rangle _{\mathrm{s}}\left| 1 \right\rangle _{\mathrm{m}}.$$

The quantum erasure works in a probabilistic way if only one POVM element ${{\hat {\mathrm \Pi}}}_{\mathrm{a}}^ +$ is considered. However, the erasure scheme can be deterministic if the other POVM element ${{\hat {\mathrm \Pi}}}_{\mathrm{a}}^ - = \hat {\Bbb I} - {{\hat {\mathrm \Pi}}}_{\mathrm{a}}^ +$ is also taken into account, in that an additional interaction between the system and the meter qubits is required. See Supplementary Note 1 for more details.

Finally, we project the system qubit onto the projector ${{\hat {\mathrm \Pi}}}_{\mathrm{s}}^\phi = \left| \phi \right\rangle \left\langle \phi \right|$, leaving the meter qubit in the state

6 $$\left| {{\Phi}} \right\rangle _{\mathrm{m}} \propto \left| 0 \right\rangle _{\mathrm{m}} + g\left\langle {\hat B\hat A} \right\rangle _{\mathrm{w}}\left| 1 \right\rangle _{\mathrm{m}},$$

where $\left\langle {\hat B\hat A} \right\rangle _{\mathrm{w}} \equiv \left\langle {\phi \left| {\hat B\hat A} \right|\psi } \right\rangle {\mathrm{/}}\left\langle {\phi |\psi } \right\rangle$ is the sequential weak value for the two non-commuting observables $\hat B$ and $\hat A$.

Since the sequential weak value $\left\langle {\hat B\hat A} \right\rangle _{\mathrm{w}}$ is registered in the meter qubit, it can be read out by obtaining certain expectation values for the meter qubit. For instance, the real part of $\left\langle {\hat B\hat A} \right\rangle _{\mathrm{w}}$ is obtained from $\left\langle {{\mathrm{{\Phi}}}\left| {\hat \sigma _x} \right|{\mathrm{{\Phi}}}} \right\rangle _{\mathrm{m}} \equiv \left( {N^ + - N^ - } \right){\mathrm{/}}\left( {N^ + + N^ - } \right)$, where *N*^+^ (*N*^−^) is the coincidence counts of *D*_1_ and *D*_2_ when the meter qubit is projected on the |+〉 (|−〉) state. The complex sequential weak value is thus given by

7 $$\left\langle {\hat B\hat A} \right\rangle _{\mathrm{w}} = \frac{1}{{2g}}\left( {\left\langle {\hat \sigma _x} \right\rangle _{\mathrm{m}} + i\left\langle {\hat \sigma _y} \right\rangle _{\mathrm{m}}} \right).$$

As our scheme combines two-photon quantum interference for the measurement interaction and sequential measurement of two non-commuting observables, it is able to demonstrate the genuine quantum nature of the sequential weak value. Moreover, unlike prior sequential weak value experiments^33,34^ in which the sequential weak values were inferred from the covariance of two meter variables, our scheme allows direct measurement of the sequential weak value. We also point out that, even though the first measurement interaction is strong, the sequential weak value for two observables can be obtained by erasing the information registered on the ancilla qubit^44^.

### Experimental sequential weak values

**Fig. 2:**
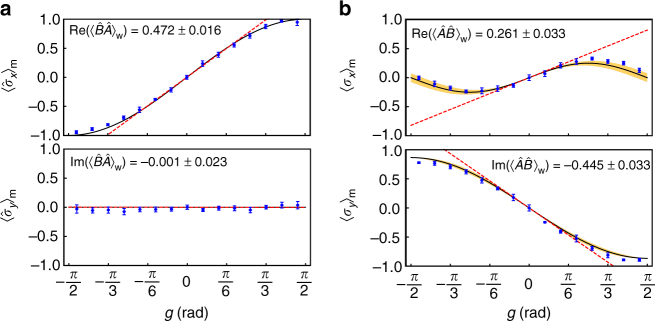
Extracting sequential weak values. The data points indicate the measured expectation values for the meter photon as a function of the weak measurement strength *g*. The system qubit is initially prepared in $\left| \psi \right\rangle _{\mathrm{s}} = \left( {\left| 0 \right\rangle - i\sqrt 3 \left| 1 \right\rangle } \right){\mathrm{/}}2$ and the final post-selective projection measurement is defined by ${{\hat {\mathrm \Pi}}}_{\mathrm{s}}^\phi = \left| - \right\rangle \left\langle - \right|$. For **a**, the two non-commuting observables are $\hat A = \left| - \right\rangle \left\langle - \right|$ and $\hat B = \left| 0 \right\rangle \left\langle 0 \right|$. For **b**, $\hat A$ and $\hat B$ are exchanged. The black solid lines are the exact theoretical curves. The dashed lines are the first-order dependence of *g* obtained from the polynomial fit to the data from which the sequential weak value is extracted. The shaded regions represent simulated errors assuming the phase instability of ±*π*/36 radians in the BD interferometer. Note that shaded regions for **a** are too narrow to be visible. The measured sequential weak values are in good agreement with the theoretical values $\langle {\hat B\hat A} \rangle _{\mathrm{w}} = 0.5$ and $\langle {\hat A\hat B} \rangle _{\mathrm{w}} = 0.250 - 0.433i$. Error bars represent one standard deviation due to Poissonian counting statistics

As described in Eq. (7), the sequential weak value $\left\langle {\hat B\hat A} \right\rangle _{\mathrm{w}}$ can be obtained by measuring the expectation values $\left\langle {\hat \sigma _x} \right\rangle _{\mathrm{m}}$ and $\left\langle {\hat \sigma _y} \right\rangle _{\mathrm{m}}$ for the meter qubit. The results of the meter qubit readout, i.e., $\left\langle {\hat \sigma _x} \right\rangle _{\mathrm{m}}$ and $\left\langle {\hat \sigma _y} \right\rangle _{\mathrm{m}}$, as a function of the measurement strength parameter *g* is shown in Fig. 2. For this measurement, the system qubit is first prepared in a specific state $\left| \psi \right\rangle _{\mathrm{s}} = \left( {\left| 0 \right\rangle - i\sqrt 3 \left| 1 \right\rangle } \right){\mathrm{/}}2$. The two incompatible observables are chosen to be $\hat A = \left| - \right\rangle \left\langle - \right|$ and $\hat B = \left| 0 \right\rangle \left\langle 0 \right|$, and the final projection basis for the system qubit is chosen to be ${{\hat {\mathrm \Pi}}}_{\mathrm{s}}^\phi = \left| - \right\rangle \left\langle - \right|$.

While it is possible to obtain the sequential weak value for a fixed *g* in the limit of $\left| g \right| \ll 1$, this may introduce systematic errors, such as, inaccurate reading of waveplate angles. To avoid this, the data set shown in Fig. 2 is fully fitted with a polynomial function of *g*. The first-order linear dependence of the polynomial fit (red dashed line) exactly corresponds to the sequential weak value. The measured values are in good agreement with the theoretical predictions.

Note that, for the sequential weak value, the measurement interaction strength *g* is very small so that the measurement process may be considered to be non-invasive. Classical intuition then may lead that the time ordering should be irrelevant to the measurement outcome^45^, i.e., $\langle {\hat A\hat B} \rangle _{\mathrm{w}} = \langle {\hat B\hat A} \rangle _{\mathrm{w}}$. However, this is not the case because $\langle {\hat A\hat B} \rangle _{\mathrm{w}} \ne \langle {\hat B\hat A} \rangle _{\mathrm{w}}$ as clearly demonstrated in Fig. 2. This result clearly distinguishes quantum measurement from classical measurement even in when the measurement strength is extremely weak so that the measurements may be considered non-perturbing^46^. It should be noted that the result $\langle {\hat A\hat B} \rangle _{\mathrm{w}} \ne \langle {\hat B\hat A} \rangle _{\mathrm{w}}$ cannot be interpreted as the consequence of the non-commuting nature of the measurement interactions. Note also that weak value measurement allows one to directly characterize quantum states^26^. We have also carried out direct quantum state tomography (d-QST) of the system qubit by using the sequential weak value^32,33^, see Methods section for details.

### Direct quantum process tomography

Identifying an unknown quantum process or operation is a crucial task in quantum information technology. We now show that the sequential weak value measurement demonstrated so far allows one to perform direct quantum process tomography (d-QPT). Typically, a quantum process $\hat {\cal E}:\hat \rho _{{\mathrm{in}}} \to \hat \rho _{{\mathrm{out}}}$ in a *d*-dimension Hilbert space is represented as $\hat {\cal E}\left( {\hat \rho _{{\mathrm{in}}}} \right) = \mathop {\sum}\nolimits_{i = 1}^{d^2} {\kern 1pt} \mathop {\sum}\nolimits_{j = 1}^{d^2} {\kern 1pt} \chi _{ij}{\hat{\mathrm E}}_i\hat \rho _{{\mathrm{in}}}{\hat{\mathrm E}}_j^{\mathrm{\dagger }}$, where $\left\{ {\hat E_i} \right\}$ is the operator basis set. The quantum process $\hat {\cal E}$ is completely characterized by matrix elements *χ*_*ij*_ of the process matrix *χ*. For a single-qubit operation, the usual choice for $\left\{ {\hat E_i} \right\}$ is the Pauli basis set $\left\{ {\hat I,\hat \sigma _x,\hat \sigma _y,\hat \sigma _z} \right\}$, but this is not a unique choice. In particular, we consider the following basis set $\left\{ {\hat S_1 = {\textstyle{1 \over {\sqrt 2 }}}\left| 0 \right\rangle \left\langle + \right|,\hat S_2 = {\textstyle{1 \over {\sqrt 2 }}}\left| 0 \right\rangle \left\langle - \right|} \right.$, $\left. {\hat S_3 = {\textstyle{1 \over {\sqrt 2 }}}\left| 1 \right\rangle \left\langle + \right|,\hat S_4 = {\textstyle{1 \over {\sqrt 2 }}}\left| 1 \right\rangle \left\langle - \right|} \right\}$, referred to as the Dirac basis following the analogy with the Dirac distribution, which characterizes a quantum state in complementary bases^26,47^. We now rewrite the quantum process in the Dirac basis as

8 $$\hat {\cal E}\left( {\hat \rho _{{\mathrm{in}}}} \right) = \mathop {\sum}\limits_{i = 1}^{d^2} {\kern 1pt} \mathop {\sum}\limits_{j = 1}^{d^2} {\kern 1pt} \chi _{ij}^S\hat S_i\hat \rho _{{\mathrm{in}}}\hat S_j,$$

where $\chi _{ij}^S$ is the *χ*-matrix elements in the Dirac basis.

**Fig. 3:**
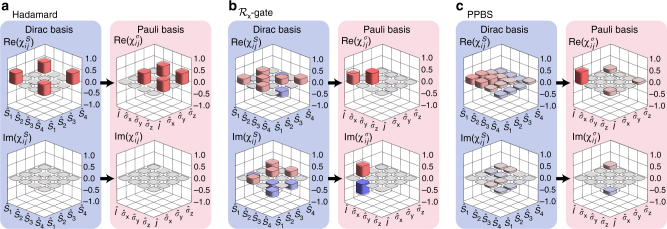
Direct-QPT via sequential weak values. The quantum operation $\hat {\cal E}$ inserted between the observables $\hat A$ and $\hat B$ can be characterized by measuring the sequential weak values. **a** $\hat {\cal E}$ is the Hadamard operation implemented by HWP set at 22.5°. **b** $\hat {\cal E}$ is the ${\cal R}_x$-gate operation implemented by QWP set at 45°, the polarization rotating operation along *x*-axis. **c** $\hat {\cal E}$ is the PPBS operation. The directly measured raw process matrices $\chi _{ij}^S$ in Dirac basis are shown at left. The corresponding process matrices $\chi _{ij}^\sigma$ in the standard Pauli basis $\{ {\hat I,\hat \sigma _x,\hat \sigma _y,\hat \sigma _z} \}$ are shown at right, where the process matrices are reconstructed to be physical matrices via the maximum likelihood estimation technique. Solid (empty) bars represent experimental (theoretical) results. The fidelities between the measured and the ideal $\chi _{ij}^\sigma$ are **a** ${\cal F} = 0.974 \pm 0.022$, **b** ${\cal F} = 0.963 \pm 0.007$ and **c** ${\cal F} = 0.973 \pm 0.007$. The errors in ${\cal F}$ are obtained by performing 500 Monte–Carlo simulation runs by taking into account of the statistical errors in measured weak values

To perform direct quantum process tomography via sequential weak value measurement in the Dirac basis, an arbitrary single-qubit quantum process $\hat {\cal E}$ is placed between the two non-commuting observables $\hat A$ and $\hat B$ as shown in Fig. 1. By choosing a specific set of the input system qubit |*ψ*〉_s_, measurement observables $\hat A$ and $\hat B$, and the final projection basis for the system qubit ${{\hat {\mathrm \Pi}}}_{\mathrm{s}}^\phi = \left| \phi \right\rangle \left\langle \phi \right|$, we are able to directly measure $\chi _{ij}^S$ from the sequential weak values, see Methods section for the specific settings. In experiment, we have tested d-QPT via sequential weak value measurement for trace-preserving and non-trace-preserving maps. The experimental process matrices are shown in Fig. 3 and they are indeed very close to the ideal process matrices, showing high fidelity of ${\cal F} > 0.96$ for all cases.

One of the key purposes of quantum process tomography in experiment is to quantitatively compare a physically implemented quantum operation with the intended ideal operation. The d-QPT via sequential weak value is uniquely well suited for this purpose over standard full quantum process tomography^48–50^. For instance, consider a trace-preserving operation such as the Hadamard gate operation. In order to judge the fidelity of the physical operation to the ideal Hadamard operation, it is not essential to know the full process matrix which may contain 16 non-zero elements. In fact, the fidelity ${\cal F}$ for Hadamard operation can be obtained with only three real or imaginary process matrix elements and is given by ${\cal F} = \sqrt {{\mathrm{Re}}\left( {\chi _{11}^S + \chi _{44}^S} \right){\mathrm{/}}2 + {\mathrm{Re}}\left( {\chi _{14}^S} \right)}$. For the ${\cal R}_x$-gate operation, the fidelity calculations requires four process matrix elements and is given by ${\cal F} = \sqrt {{\mathrm{Re}}\left( {2\chi _{13}^S - 2\chi _{24}^S + 1} \right){\mathrm{/}}4 + {\mathrm{Im}}\left( {\chi _{12}^S - \chi _{34}^S} \right)}$. ${\cal R}_x$-gate operation is a polarization rotating operation along *x*-axis, that is defined as ${\cal R}_x\left| 0 \right\rangle \to \left( {\left| 0 \right\rangle + i\left| 1 \right\rangle } \right){\mathrm{/}}\sqrt 2$, ${\cal R}_x\left| 1 \right\rangle \to \left( {\left| 0 \right\rangle - i\left| 1 \right\rangle } \right){\mathrm{/}}\sqrt 2$. See Supplementary Note 3 for details. The resulting fidelity values are ${\cal F} = 0.973 \pm 0.023$ for the Hadamard operation and ${\cal F} = 0.970 \pm 0.011$ for the ${\cal R}_x$-gate operation and these fidelity values are indeed in good agreement with the fidelity values from the full quantum process tomography.

We now compare d-QPT-based fidelity estimation with the compressive-sensing quantum process tomography (cs-QPT)^40,41^. Even though our d-QPT method requires additional resource qubits, both d-QPT and cs-QPT are equally more efficient than the standard full QPT in the sense that the fidelity of a quantum process can be quantified without complete measurements. However, the difference is in the requirement of prior knowledge and assumptions. In cs-QPT, an unknown quantum process can be accurately quantified with the assumption that it can be represented by a sparse or low-rank matrix. Thus, if the sparsity assumption is not valid, cs-QPT would give incorrect fidelity estimation. In contrast, d-QPT-based fidelity estimation always produces faithful results with the prior knowledge of the target operation.

It is also interesting to consider the possibility of combining cs-QPT and d-QPT based on sequential weak values for more efficient characterization of a quantum process. In cs-QPT, incomplete sets of experimentally measured outcomes with randomly chosen input states and projective measurement settings are fed in to the compressive-sensing algorithm. Interestingly, we have found that certain sequential weak values corresponding to specific process matrix elements in Dirac basis could be used as the input to cs-QPT for more efficient characterization of a quantum process. More details of the cs-QPT with sequential weak values may be found in Supplementary Note 3. For instance, one first measure an incomplete set of sequential weak values (or randomly chosen process matrix elements in Dirac basis) for an unknown quantum process. The results are then fed into cs-QPT and the reliability of the cs-QPT is evaluated accurately via d-QPT fidelity estimation, which may require measuring a few more sequential weak values. This process may be repeated until a certain reliability bound is reached.

## Discussion

We have demonstrated measurement of the sequential weak values of two incompatible observables. By making use of two-photon quantum interference for the measurement interaction, our measurement scheme can be viewed as an unambiguous quantum physical implementation of sequential weak measurement. The genuine quantum nature of the sequential weak value for two incompatible observables reported in this work will be instrumental in rigorous tests quantum contextuality^36,37^, macroscopic realism^38,39^, uncertainty relations^51^, measurement induced geometric phase^52^, etc. Furthermore, our sequential measurement scheme is in principle nondestructive and its measurement strength is controllable. We anticipate such features will be useful for quantum feedback control via measurement^53,54^. We have also demonstrated for the first time that the sequential weak value measurement can be used to perform direct quantum process tomography of a quantum channel. By combining the idea of compressing sensing with d-QPT fidelity estimation, an efficient strategy for characterizing an unknown quantum process can be established.

## Methods

### Photon pair source

The two single-photons in the experiment are generated via ultrafast-pumped spontaneous parametric down-conversion (SPDC) in a 1 mm thick type-II BBO crystal in the beamlike configuration. The central wavelengths of the pump photon and the SPDC photons are 390 nm and 780 nm, respectively. The SPDC photons are collected into a single-mode fiber via interference filters having 3 nm full-width at half-maximum bandwidth.

### Details for direct-QST

To directly measure elements of the density matrix for the system qubit, the sequential weak values are measured with a specific setting for the observables $\hat A$ and $\hat B$ and the post-selection projection ${{\hat {\mathrm \Pi}}}_{\mathrm{s}}^\phi = \left| \phi \right\rangle \left\langle \phi \right|$. The sequential weak value for the system qubit having the density matrix $\hat \rho _{{\mathrm{in}}}$ is given as $\left\langle {\hat B\hat A} \right\rangle _{\mathrm{w}} = {\mathrm{Tr}}[ {{{\hat {\mathrm \Pi}}}_{\mathrm{s}}^\phi \hat B\hat A\hat \rho _{{\mathrm{in}}}} ]{\mathrm{/}}p$, where $p = {\mathrm{Tr}}\left[ {{{\hat {\mathrm \Pi}}}_{\mathrm{s}}{\kern 1pt} \hat \rho _{{\mathrm{in}}}} \right]$ is the post-selection probability. For simplicity, let us denote {|*a*_1_〉 = |0〉, |*a*_2_〉 = |1〉} and {|*b*_1_〉 = |+〉, |*b*_2_〉 = |−〉}. The observables are $\hat A = \left| {a_m} \right\rangle \left\langle {a_m} \right|$ and $\hat B = \left| {b_1} \right\rangle \left\langle {b_1} \right|$. The projector is given by ${{\hat {\mathrm \Pi}}}_{\mathrm{s}} = \left| {a_n} \right\rangle \left\langle {a_n} \right|$. Then, the elements of $\hat \rho _{{\mathrm{in}}}$ can be directly obtained from the sequential weak values, i.e., $\rho _{mn} = \left\langle {\hat B\hat A} \right\rangle _{\mathrm{w}}^{mn} \times 2p^n$. We have tested d-QST with sequential weak value measurement and the results are in good agreement with the ideal target density matrix with fidelity better than ${\cal F} = 0.994 \pm 0.008$. See Supplementary Note 2 for more details and experimental data.

### Details for direct-QPT

For a single-qubit quantum process, there are 16 elements for the *χ*-matrix and it is possible to access these matrix elements directly via sequential weak value measurement with specific choices of settings. We first define $\hat \rho _{{\mathrm{in}}}^{n\prime } = \left| {a_{n\prime }} \right\rangle \left\langle {a_{n\prime }} \right|$, $\hat A^k = \left| {b_k} \right\rangle \left\langle {b_k} \right|$, $\hat B^n = \left| {a_n} \right\rangle \left\langle {a_n} \right|$, and ${{\hat {\mathrm \Pi}}}_{\mathrm{s}}^{k\prime } = \left| {b_{k\prime }} \right\rangle \left\langle {b_{k\prime }} \right|$. The sequential weak values are corresponding to the process matrix elements as $\chi _{ij}^S = \chi _{knk{\prime}n{\prime}}^S = \left\langle {\hat B^n\hat A^k} \right\rangle _{\mathrm{w}}^{n{\prime}k{\prime}} \times 2p^{n{\prime}k{\prime}} \times \left( { - 1} \right)^{\delta _{k2}\delta _{n\prime 2}}\left( { - 1} \right)^{\delta _{k\prime 2}\delta _{n2}}$, where $p^{n\prime k\prime } = {\mathrm{Tr}}[ {{{\hat {\mathrm \Pi}}}_{\mathrm{s}}^{k\prime }{\cal E}( {\hat \rho _{{\mathrm{in}}}^{n\prime }} )} ]$ is the post-selection probability, *δ*_*i*2_ is the Kronecker delta, and the subscripts *kn* and *k*ʹ*n*ʹ are the binary number representations of *i* and *j*, respectively. See Supplementary Note 3 for more details.

### Data availability

The data sets analyzed during this study are available from the corresponding author on reasonable request.

## Electronic supplementary material

Supplementary Information
