# Publisher Correction: Direct quantum process tomography via measuring sequential weak values of incompatible observables

**DOI:** 10.1038/s41467-018-03305-w

**Published:** 2018-02-22

**Authors:** Yosep Kim, Yong-Su Kim, Sang-Yun Lee, Sang-Wook Han, Sung Moon, Yoon-Ho Kim, Young-Wook Cho

**Affiliations:** 1 Department of Physics, Pohang University of Science and Technology (POSTECH), 37673 Pohang, Korea; 2 Center for Quantum Information, Korea Institute of Science and Technology (KIST), 02792 Seoul, Korea

Correction to: *Nature Communications* 10.1038/s41467-017-02511-2, published online 15 January 2018

In the original version of this Article, the penultimate sentence of the second paragraph of the “Schematic and theory” section of the Results originally incorrectly read “For instance, choosing θ_A_ = π/4, 0, −π/8 and π/8 rotates the bases $$\left| 0 \right\rangle$$, $$\left| 1 \right\rangle$$, $$\left| + \right\rangle$$, and $$\left| - \right\rangle$$ into $$\left| 1 \right\rangle$$, thereby implementing the observable $$\hat A = \left| 0 \right\rangle \left\langle 0 \right|$$, $$\left| 1 \right\rangle$$$$\left\langle 1 \right|$$, $$\left| + \right\rangle$$$$\left\langle + \right|$$ and, respectively.” In the corrected version, “$$\left| - \right\rangle$$$$\left\langle - \right|$$” has been added before “, respectively”. This error has been corrected in both the PDF and HTML versions of the Article.

