# Supplementary material for: Direct quantum process tomography via measuring sequential weak values of incompatible observables
Source: Nat Commun. 2018 Jan 15;9:192. doi: 10.1038/s41467-017-02511-2 (PMC5768737; doi:10.1038/s41467-017-02511-2)
Supplement: Supplementary file 1 — Supplementary Information [file 41467_2017_2511_MOESM1_ESM.pdf]

SUPPLEMENTARY NOTE 1 – DETAILS ON THE SEQUENTIAL WEAK VALUE MEASUREMENT SCHEME

In this note, we give more details on the schematic and the theory for the sequential weak value measurement for observable $\hat{A}$ and $\hat{B}$. Note that the schematic is based on the proposal of realizing von-Neumann measurement with variable measurement strength via quantum erasure in Ref. [1]. As in the main text, we consider three qubits: a system qubit $|\psi\rangle_{\mathrm{s}}$, an ancillary qubit $|\psi\rangle_{\mathrm{a}}$, and a meter qubit $|\Phi\rangle_{\mathrm{m}}$, where the system is a quantum state to be measured and the meter is used to register the measurement outcome. The ancilla is used to temporarily register the measurement outcome for the first observable $\hat{A}$, the registered information of which is erased finally to leave all the measurement information in the meter qubit.

Initially, the total quantum state is prepared in $|\psi\rangle_{\mathrm{s}}|0\rangle_{\mathrm{a}}|0\rangle_{\mathrm{m}}$. In our scheme, the first interaction for observable $\hat{A}$ is a controlled-$\hat{\sigma}_x$ (CNOT) type interaction between system and ancillary qubits. Here $\hat{\sigma}_x, \hat{\sigma}_y$ and $\hat{\sigma}_z$ stand for Pauli operators. The CNOT type interaction can be described as a unitary operation $\hat{U}_{\mathrm{A}} = (\hat{\mathbb{I}} - \hat{A}) \otimes \hat{\mathbb{I}} + \hat{A} \otimes \hat{\sigma}_x$ on the system and the ancilla qubits, where $\hat{\mathbb{I}}$ is the identity operation. After applying $\hat{U}_{\mathrm{A}}$, the total state evolves to

$$\hat{U}_{\mathrm{A}}|\psi\rangle_{\mathrm{s}}|0\rangle_{\mathrm{a}} \otimes |0\rangle_{\mathrm{m}} = |\psi\rangle_{\mathrm{s}}|0\rangle_{\mathrm{a}}|0\rangle_{\mathrm{m}} - \hat{A}|\psi\rangle_{\mathrm{s}}|0\rangle_{\mathrm{a}}|0\rangle_{\mathrm{m}} + \hat{A}|\psi\rangle_{\mathrm{s}}|1\rangle_{\mathrm{a}}|0\rangle_{\mathrm{m}}. \tag{1}$$

Next, we consider the measurement interaction for the observable $\hat{B}$. That is the controlled-controlled-σ_z (CCZ) operation with rotating operations on the meter qubit, where the unitary evolution for the second interaction is given as

$$\hat{U}_{\mathrm{B}} = \hat{\mathbb{I}} \otimes \hat{\Pi}^0_{\mathrm{a}} \otimes \hat{\mathbb{I}} + (\hat{\mathbb{I}} - \hat{B}) \otimes \hat{\Pi}^1_{\mathrm{a}} \otimes \hat{\mathbb{I}} + \hat{B} \otimes \hat{\Pi}^1_{\mathrm{a}} \otimes \hat{R}^{-1}\left(\frac{g}{4}\right)\hat{\sigma}_z \hat{R}\left(\frac{g}{4}\right), \tag{2}$$

where $\hat{\Pi}^i_{\mathrm{a}} = |i\rangle\langle i|$ is the projector on the ancillary qubit, the measurement strength is adjusted by g and the rotating matrix on meter qubit is givens as

$$\hat{R}(\theta) = \begin{pmatrix} \cos(2\theta) & \sin(2\theta) \\ \sin(2\theta) & -\cos(2\theta) \end{pmatrix}. \tag{3}$$

The total states after applying $\hat{U}_{\mathrm{B}}$ is given as

$$\hat{U}_{\mathrm{B}}(\hat{U}_{\mathrm{A}}|\psi\rangle_{\mathrm{s}}|0\rangle_{\mathrm{a}} \otimes |0\rangle_{\mathrm{m}}) \approx |\psi\rangle_{\mathrm{s}}|0\rangle_{\mathrm{a}}|0\rangle_{\mathrm{m}} - \hat{A}|\psi\rangle_{\mathrm{s}}|0\rangle_{\mathrm{a}}|0\rangle_{\mathrm{m}} + \hat{A}|\psi\rangle_{\mathrm{s}}|1\rangle_{\mathrm{a}}|0\rangle_{\mathrm{m}} + g\hat{B}\hat{A}|\psi\rangle_{\mathrm{s}}|1\rangle_{\mathrm{a}}|1\rangle_{\mathrm{m}}, \tag{4}$$

where we have used an approximation up to the first order of g with the assumption of the weak measurement condition $g \ll 1$. From the last term it is clear that the action of the sequential observables $\hat{B}\hat{A}$ is registered on the meter qubit, but the superfluous second and third terms still remain. To remove these terms, we can consider to apply a projector on the ancilla qubit $\hat{\Pi}^+_{\mathrm{a}} = |+\rangle\langle+|$ where $|+\rangle \equiv (|0\rangle + |1\rangle)/\sqrt{2}$ (The projector $\hat{\Pi}^+_{\mathrm{a}}$ can be viewed as a quantum erasure as it erases the measurement results registered in the ancilla qubit [1]). Then, the joint state of system and meter qubits after projecting ancilla qubit with $\hat{\Pi}^+_{\mathrm{a}}$ is given as

$$|\psi\rangle_{\mathrm{s}}|0\rangle_{\mathrm{m}} + g\hat{B}\hat{A}|\psi\rangle_{\mathrm{s}}|1\rangle_{\mathrm{m}}. \tag{5}$$

Finally, the system qubit is projected on $|\phi\rangle_{\mathrm{s}}$ with a projector $\hat{\Pi}^\phi_{\mathrm{s}} = |\phi\rangle\langle\phi|$. Then, the remained meter state is given as

$$|\Phi\rangle_{\mathrm{m}} \propto |0\rangle_{\mathrm{m}} + g\langle\hat{B}\hat{A}\rangle_{\mathrm{w}}|1\rangle_{\mathrm{m}}, \tag{6}$$

where $\langle\hat{B}\hat{A}\rangle_{\mathrm{w}} \equiv \langle\phi|\hat{B}\hat{A}|\psi\rangle/\langle\phi|\psi\rangle$ is the sequential weak value for observables $\hat{A}$ and $\hat{B}$. The sequential weak value is registered in the amplitude of meter state, and it thus can be extracted by measuring the typical expectation values of Pauli observables, such as $\hat{\sigma}_x$ and $\hat{\sigma}_y$:

$$\mathrm{Re}\{\langle\hat{B}\hat{A}\rangle_{\mathrm{w}}\} = \frac{1}{2g}\,{}_{\mathrm{m}}\langle\Phi|\hat{\sigma}_x|\Phi\rangle_{\mathrm{m}} = \frac{1}{2g}\langle\hat{\sigma}_x\rangle_{\mathrm{m}}, \tag{7}$$

$$\mathrm{Im}\{\langle\hat{B}\hat{A}\rangle_{\mathrm{w}}\} = \frac{1}{2g}\,{}_{\mathrm{m}}\langle\Phi|\hat{\sigma}_y|\Phi\rangle_{\mathrm{m}} = \frac{1}{2g}\langle\hat{\sigma}_y\rangle_{\mathrm{m}}. \tag{8}$$

We further note that the quantum erasure is probabilistic as we only consider one POVM element $\hat{\Pi}^+_{\mathrm{a}}$. However, the eraser scheme can be deterministic if an additional interaction between the system and the meter qubits is allowed.

Let us consider the other POVM element $\hat{\Pi}_{\rm a}^{-} = \hat{\mathbb{I}} - \hat{\Pi}_{\rm a}^{+} = |-\rangle\langle -|$. If the ancillary state is projected by $\hat{\Pi}_{\rm a}^{-}$, the system and meter state is given as

$$(\hat{\mathbb{I}} - 2\hat{A})|\psi\rangle_{\rm s}|0\rangle_{\rm m} - g\hat{B}\hat{A}|\psi\rangle_{\rm s}|1\rangle_{\rm m}. \tag{9}$$

An additional feed-forward interaction, $\hat{U}_{\rm C} = (\hat{\mathbb{I}} - 2\hat{A})^{-1} \otimes \hat{\Pi}_{\rm m}^{0} - \hat{\mathbb{I}} \otimes \hat{\Pi}_{\rm m}^{1}$, is applied to the system and the meter qubits. After projecting the system state with $\hat{\Pi}_{\rm s}^{\phi}$, the meter state is given as

$$|\Phi\rangle_{\rm m} \propto |0\rangle_{\rm m} + g\langle\hat{B}\hat{A}\rangle_{\rm w}|1\rangle_{\rm m}. \tag{10}$$

Therefore, the sequential weak value can be deterministically extracted from both POVMs with Supplementary Equations (7-8).

SUPPLEMENTARY NOTE 2 – DIRECT QUANTUM STATE TOMOGRAPHY VIA SEQUENTIAL WEAK VALUES

A 2×2 input density matrix $\hat{\rho}_{\rm in}$ in H/V basis is represented as

$$\hat{\rho}_{\rm in} = \begin{pmatrix} \rho_{11} & \rho_{12} \\ \rho_{21} & \rho_{22} \end{pmatrix}. \tag{11}$$

Each element of the density matrix is described as $\rho_{mn} = {\rm Tr}[|a_m\rangle\langle a_n|\rho_{\rm in}]$ where $|a_1\rangle = |{\rm H}\rangle$ and $|a_2\rangle = |{\rm V}\rangle$, i.e. ρ_{mn} can be obtained from the expectation value of $\hat{\rho}_{\rm in}$ for the $|a_m\rangle\langle a_n|$ operator. In cases of off-diagonal elements, the operators are non-Hermitian operator which gives complex value as the expectation value.

The sequential weak values with incompatible observables allow to implement the non-Hermitian operator and measure the complex expectation value. The weak value for an input density matrix $\hat{\rho}_{\rm in}$ is given as

$$\langle\hat{B}\hat{A}\rangle_{\rm w} = \frac{{\rm Tr}[\hat{\Pi}_{\rm s}^{\phi}\hat{B}\hat{A}\hat{\rho}_{\rm in}]}{p}, \tag{12}$$

where $p = {\rm Tr}[\hat{\Pi}_{\rm s}^{\phi}\hat{\rho}_{\rm in}]$ is the post-selection probability. Therefore, the elements of density matrix can be measured directly by setting observables and post-selection to be $|a_m\rangle\langle a_n|$.

For the direct quantum state tomography (d-QST), the observables $\hat{A}$ and $\hat{B}$ and post-selection $\hat{\Pi}_{\rm s}^{\phi}$ are set as $\hat{A} = \{|a_1\rangle\langle a_1|, |a_2\rangle\langle a_2|\}$, $\hat{B} = \{|b_1\rangle\langle b_1|, |b_2\rangle\langle b_2|\}$ and $\hat{\Pi}_{\rm s}^{\phi} = \{|a_1\rangle\langle a_1|, |a_2\rangle\langle a_2|\}$. The $|a_i\rangle$ and $|b_i\rangle$ are orthonormal basis and mutually unbiased $|\langle a_i|b_j\rangle| = 1/\sqrt{2}$ [2]. In particular, for H/V basis, observables are set as $|a_1\rangle = |{\rm H}\rangle$, $|a_2\rangle = |{\rm V}\rangle$, $|b_1\rangle = |{\rm D}\rangle$, and $|b_2\rangle = |{\rm A}\rangle$. And, let us fix $\hat{B} = |b_1\rangle\langle b_1|$, and consider $\hat{A} = |a_m\rangle\langle a_m|$ and $\hat{\Pi}_{\rm s}^{n} = |a_n\rangle\langle a_n|$. With this setting, the density matrix element can be directly measured from the sequential weak value $\langle\hat{B}\hat{A}\rangle_{\rm w}^{mn}$ and post selection probability $p^n = {\rm Tr}[\hat{\Pi}_{\rm s}^{n}\hat{\rho}_{\rm in}]$,

$$\rho_{mn} = \langle\hat{B}\hat{A}\rangle_{\rm w}^{mn} \times 2p^n. \tag{13}$$

In addition, the density matrix in different basis can be measured by setting the observables and post-selection in other ways. For example, the set of $|a_1\rangle = |{\rm D}\rangle$, $|a_2\rangle = |{\rm A}\rangle$, $|b_1\rangle = |{\rm H}\rangle$, and $|b_2\rangle = |{\rm V}\rangle$ allow to do d-QST in D/A basis.

We test the d-QST for a pure state $|\psi_{\rm in}\rangle = (|{\rm H}\rangle - i\sqrt{3}|{\rm V}\rangle)/2$ with our sequential weak value method, see Supplementary Figure **1**. By setting the $|a_i\rangle, |b_j\rangle$ for d-QST in D/A basis, we measure the sequential weak values corresponding to each density matrix elements. **a** ρ_{11}, **b** ρ_{12}, **c** ρ_{21} and **d** ρ_{22} in D/A basis. And, the p^n is estimated from the ratio between coincidence counts in cases of $\hat{\Pi}_{\rm s}^{1} = |{\rm D}\rangle\langle{\rm D}|$ and $\hat{\Pi}_{\rm s}^{2} = |{\rm A}\rangle\langle{\rm A}|$. Because the $|{\rm D}\rangle\langle{\rm D}|$ and $|{\rm A}\rangle\langle{\rm A}|$ make complete set, the sum of post-selection probability is unity. So, the probability can be obtained from the coincidence counts ratio with the condition that the sum is unity. The **e** shows the result of d-QST with maximum likelihood method. The directly measured density matrix shows an excellent agreement with the ideal density matrix by the fidelity of $\mathcal{F} = 0.994 \pm 0.008$.

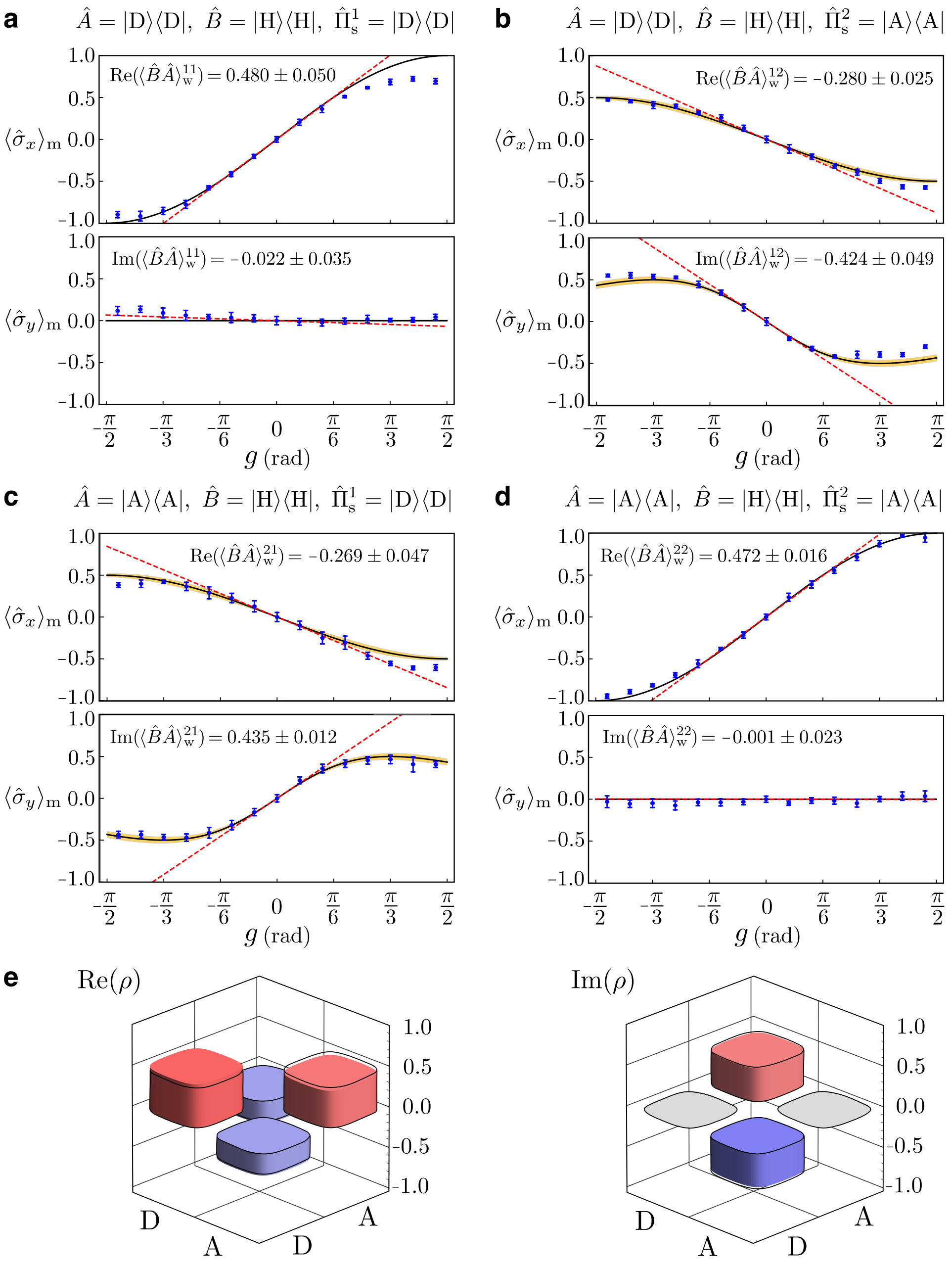

Supplementary Figure 1. **Direct quantum state tomography via sequential weak value.** To measure the elements of 2×2 density matrix directly, sequential weak values are measured with four observable sets. The observable sets consisting of two incompatible observables and post-selection $\{\hat{A}, \hat{B}, \hat{\Pi}_{\mathrm{s}}^{\phi}\}$ are prepared as **a** $\{|\mathrm{D}\rangle\langle\mathrm{D}|, |\mathrm{H}\rangle\langle\mathrm{H}|, |\mathrm{D}\rangle\langle\mathrm{D}|\}$, **b** $\{|\mathrm{D}\rangle\langle\mathrm{D}|, |\mathrm{H}\rangle\langle\mathrm{H}|, |\mathrm{A}\rangle\langle\mathrm{A}|\}$, **c** $\{|\mathrm{A}\rangle\langle\mathrm{A}|, |\mathrm{H}\rangle\langle\mathrm{H}|, |\mathrm{D}\rangle\langle\mathrm{D}|\}$ and **d** $\{|\mathrm{A}\rangle\langle\mathrm{A}|, |\mathrm{H}\rangle\langle\mathrm{H}|, |\mathrm{A}\rangle\langle\mathrm{A}|\}$ for elements of the density matrix ρ_{11}, ρ_{12}, ρ_{21} and ρ_{22}, respectively. The initial state or tomography target state is $|\psi_{\mathrm{in}}\rangle = (|\mathrm{H}\rangle - i\sqrt{3}|\mathrm{V}\rangle)/2$. From the (a-d), sequential weak values are extracted for direct quantum state tomography. The data points indicate the measured expectation values for the meter photon as a function of the measurement interaction strength g. The black solid lines are the exact theoretical expectations, error bars in **a**–**d** represent one standard deviation due to Poissonian counting statistics, the shaded regions represents simulated errors assuming the phase instability of $\pm\pi/36$ radians in the beam displacer (BD) interferometer. Note that shaded regions for **a** and **d** are too small to be visible. The dashed lines are the first order dependence of g obtained from the polynomial fit with data points, from which the weak values can be extracted. The measured sequential weak values are **a** $\langle\hat{B}\hat{A}\rangle_{\mathrm{w}}^{11} = (0.480\pm0.050)+(-0.022\pm0.035)i$, **b** $\langle\hat{B}\hat{A}\rangle_{\mathrm{w}}^{12} = (-0.280\pm0.025)+(-0.424\pm0.049)i$, **c** $\langle\hat{B}\hat{A}\rangle_{\mathrm{w}}^{21} = (-0.269\pm0.047)+(0.435\pm0.012)i$ and **d** $\langle\hat{B}\hat{A}\rangle_{\mathrm{w}}^{22} = (0.472 \pm 0.016) + (-0.001 \pm 0.023)i$. And, from the ratio of coincidence counts in two projectors $|\mathrm{D}\rangle\langle\mathrm{D}|$ and $|\mathrm{A}\rangle\langle\mathrm{A}|$, p^1 and p^2 are estimated as 0.483 and 0.516 for $\hat{\Pi}_{\mathrm{s}}^1 = |\mathrm{D}\rangle\langle\mathrm{D}|$ and $\hat{\Pi}_{\mathrm{s}}^2 = |\mathrm{A}\rangle\langle\mathrm{A}|$, respectively. With maximum likelihood (MLH) method, the reconstructed density matrix shows an excellent agreement with the ideal density matrix by the fidelity of $\mathcal{F} = 0.994 \pm 0.008$, see **e**. The color filled bars show experimental datas with MLH and the solid lines indicate the ideal values.

SUPPLEMENTARY NOTE 3 – DIRECT QUANTUM PROCESS TOMOGRAPHY VIA SEQUENTIAL WEAK VALUES

Quantum process tomography in Dirac basis

A quantum process $\mathcal{E}(\hat{\rho}_{\text{in}})$ in d-dimension Hilbert space can be represented as

$$\mathcal{E}(\hat{\rho}_{\text{in}}) = \sum_{i=1}^{d^2}\sum_{j=1}^{d^2} \chi_{ij} \hat{\text{E}}_i \hat{\rho}_{\text{in}} \hat{\text{E}}_j^\dagger, \tag{14}$$

where $\{\hat{\text{E}}_i\}$ is an operator basis set and the quantum process is fully characterized by the process matrix elements χ_{ij}. A standard choice of $\{\hat{\text{E}}_i\}$ is the Pauli basis set $\{\hat{I}, \hat{\sigma}_x, \hat{\sigma}_y, \hat{\sigma}_z\}$ or a Kraus operator basis set $\{\hat{\text{E}}_i \equiv |a_m\rangle\langle a_n|\}$, where $\{|a_k\rangle\}$ is an orthonormal state basis set and the index i is represented as new indexes $\{m, n\}$ as $i = (m-1)d + n$. We consider the Kraus basis set, and after changing the indexes i and j into $\{m, n\}$ and $\{m', n'\}$ the quantum process is then rewritten as

$$\mathcal{E}(\hat{\rho}_{\text{in}}) = \sum_{m=1}^{d}\sum_{n=1}^{d}\sum_{m'=1}^{d}\sum_{n'=1}^{d} \chi_{mn,m'n'} |a_m\rangle\langle a_n|\hat{\rho}_{\text{in}}|a_{n'}\rangle\langle a_{m'}| \tag{15}$$

We introduce another orthonormal state basis $\{|b_l\rangle\}$, which is a complementary basis set to $\{|a_k\rangle\}$, satisfying

$$\langle a_k|b_l\rangle = \frac{1}{\sqrt{d}} \exp\left[i\frac{2\pi}{d}(l-1)(k-1)\right]. \tag{16}$$

Thus, one can recognize $\{|b_l\rangle\}$ is mathematically the discrete Fourier transform of $\{|a_k\rangle\}$:

$$\begin{aligned} |b_l\rangle &= \frac{1}{\sqrt{d}} \sum_{k=1}^{d} \exp\left[+i\frac{2\pi}{d}(l-1)(k-1)\right] |a_k\rangle, \\ |a_l\rangle &= \frac{1}{\sqrt{d}} \sum_{k=1}^{d} \exp\left[-i\frac{2\pi}{d}(l-1)(k-1)\right] |b_k\rangle. \end{aligned} \tag{17}$$

After substituting the relation of Supplementary Equation (17) into Supplementary Equation (15), then we have

$$\begin{aligned} \mathcal{E}(\hat{\rho}_{\text{in}}) &= \frac{1}{d} \sum_{m=1}^{d}\sum_{n=1}^{d}\sum_{m'=1}^{d}\sum_{n'=1}^{d}\sum_{k=1}^{d}\sum_{k'=1}^{d} \chi_{mn,m'n'} \exp\left[i\frac{2\pi}{d}\{(n-1)(k-1) + (m'-1)(k'-1)\}\right] |a_m\rangle\langle b_k|\hat{\rho}_{\text{in}}|a_{n'}\rangle\langle b_{k'}| \\ &= \sum_{m=1}^{d}\sum_{n=1}^{d}\sum_{m'=1}^{d}\sum_{n'=1}^{d}\sum_{k=1}^{d}\sum_{k'=1}^{d} \chi_{mn,m'n'} \exp\left[i\frac{2\pi}{d}\{(n-1)(k-1) + (m'-1)(k'-1)\}\right] \hat{S}_i \hat{\rho}_{\text{in}} \hat{S}_j, \end{aligned} \tag{18}$$

where we have defined a new operator basis

$$\hat{S}_i = \frac{1}{\sqrt{d}} |a_m\rangle\langle b_k|, \text{ where } i = (m-1)d + k, \tag{19}$$

$$\hat{S}_j = \frac{1}{\sqrt{d}} |a_{n'}\rangle\langle b_{k'}|, \text{ where } j = (n'-1)d + k'. \tag{20}$$

Supplementary Equation (18) is further simplified by substituting

$$\chi^S_{km,k'n'} = \sum_{n=1}^{d}\sum_{m'=1}^{d} \chi_{mn,m'n'} \exp\left[i\frac{2\pi}{d}\{(n-1)(k-1) + (m'-1)(k'-1)\}\right]. \tag{21}$$

Finally, we have

$$\begin{aligned} \mathcal{E}(\hat{\rho}_{\text{in}}) &= \sum_{k=1}^{d}\sum_{m=1}^{d}\sum_{k'=1}^{d}\sum_{n'=1}^{d} \chi^S_{km,k'n'} \hat{S}_i \hat{\rho}_{\text{in}} \hat{S}_j & (22) \\ &= \sum_{i=1}^{d^2}\sum_{j=1}^{d^2} \chi^S_{ij} \hat{S}_i \hat{\rho}_{\text{in}} \hat{S}_j, & (23) \end{aligned}$$

where kn and $k'n'$ are the binary number representations of i and j, respectively. We call the basis $\{\hat{S}_i\}$ as *Dirac basis* following the analogy with the Dirac distribution which is used to characterize a quantum state in the complementary basis [2, 3]. In our direct quantum process tomography (d-QPT) scheme, the process matrix elements χ^S_{ij} is directly measured from the sequential weak values.

Direct quantum process tomography via sequential weak values

In this subsection, we elaborate how our sequential weak value measurement scheme can be used for characterizing a unknown quantum process in Dirac basis. We consider a quantum process in the qubit Hilbert space ($d=2$) and two complementary basis sets : $\{|a_1\rangle = |0\rangle, |a_2\rangle = |1\rangle\}$ and $\{|b_1\rangle = |+\rangle, |b_2\rangle = |-\rangle\}$. The initial input state is prepared in a pure state $\hat{\rho}^{n'}_{\mathrm{in}} = |a_{n'}\rangle\langle a_{n'}|$, the first observable $\hat{A}$ is set as $\hat{A}^k = |b_k\rangle\langle b_k|$, an arbitrary quantum process $\mathcal{E}(\cdot)$ is placed between observables $\hat{A}$ and $\hat{B}$, the second observable $\hat{B}$ is set as $\hat{B}^n = |a_n\rangle\langle a_n|$, and finally we consider a projector $\hat{\Pi}^{k'}_{\mathrm{s}} = |b_{k'}\rangle\langle b_{k'}|$. With these settings, the sequential weak value is given as

$$\langle \hat{B}^n \hat{A}^k \rangle^{n'k'}_{\mathrm{w}} = \frac{\mathrm{Tr}\left[\hat{\Pi}^{k'}_{\mathrm{s}} \hat{B}^n \mathcal{E}(\hat{A}^k \hat{\rho}^{n'}_{\mathrm{in}})\right]}{p^{n'k'}}, \tag{24}$$

where

$$p^{n'k'} = \mathrm{Tr}\left[\hat{\Pi}^{k'}_{\mathrm{s}} \mathcal{E}(\hat{\rho}^{n'}_{\mathrm{in}})\right] \tag{25}$$

is the post-selection probability.

Let us calculate the denominator of Supplementary Equation (24) explicitly. Using Supplementary Equation (22), we have

$$\begin{aligned}
\mathcal{E}(\hat{A}^k \hat{\rho}^{n'}_{\mathrm{in}}) &= \sum_{\alpha=1}^{2}\sum_{\beta=1}^{2}\sum_{\alpha'=1}^{2}\sum_{\beta'=1}^{2} \chi^S_{\alpha\beta,\alpha'\beta'} \frac{1}{\sqrt{2}} |a_\alpha\rangle\langle b_\beta| \cdot \hat{A}^k \hat{\rho}^{n'}_{\mathrm{in}} \cdot \frac{1}{\sqrt{2}} |a_{\alpha'}\rangle\langle b_{\beta'}| \\
&= \sum_{\alpha=1}^{2}\sum_{\beta=1}^{2}\sum_{\alpha'=1}^{2}\sum_{\beta'=1}^{2} \chi^S_{\alpha\beta,\alpha'\beta'} \frac{1}{2} |a_\alpha\rangle\langle b_\beta| \cdot |b_k\rangle\langle b_k| \cdot |a_{n'}\rangle\langle a_{n'}| \cdot |a_{\alpha'}\rangle\langle b_{\beta'}| \\
&= \sum_{\alpha=1}^{2}\sum_{\alpha'=1}^{2}\sum_{\beta'=1}^{2} \chi^S_{\alpha k,\alpha'\beta'} \frac{(-1)^{\delta_{k2}\delta_{n'2}}}{2\sqrt{2}} |a_\alpha\rangle\langle a_{n'}| \cdot |a_{\alpha'}\rangle\langle b_{\beta'}|,
\end{aligned} \tag{26}$$

where we have used $\langle a_i | b_j \rangle = (-1)^{\delta_{i2}\delta_{j2}}/\sqrt{2}$ and δ_{i2} is the Kronecker delta. Likewise, we calculate further as

$$\begin{aligned}
\hat{\Pi}^{k'}_{\mathrm{s}} \hat{B}^n \mathcal{E}(\hat{A}^k \hat{\rho}^{n'}_{\mathrm{in}}) &= \sum_{\alpha=1}^{2}\sum_{\alpha'=1}^{2}\sum_{\beta'=1}^{2} \chi^S_{\alpha k,\alpha'\beta'} \frac{(-1)^{\delta_{k2}\delta_{n'2}}}{2\sqrt{2}} |b_{k'}\rangle\langle b_{k'}| \cdot |a_n\rangle\langle a_n| \cdot |a_\alpha\rangle\langle a_{n'}| \cdot |a_{\alpha'}\rangle\langle b_{\beta'}| \\
&= \sum_{\beta'=1}^{2} \chi^S_{nk,n'\beta'} \frac{(-1)^{\delta_{k2}\delta_{n'2}}(-1)^{\delta_{k'2}\delta_{n2}}}{4} |b_{k'}\rangle\langle b_{\beta'}|.
\end{aligned} \tag{27}$$

Then, we finally get

$$\mathrm{Tr}\left[\hat{\Pi}^{k'}_{\mathrm{s}} \hat{B}^n \mathcal{E}(\hat{A}^k \hat{\rho}^{n'}_{\mathrm{in}})\right] = \chi^S_{nk,n'k'} \frac{(-1)^{\delta_{k2}\delta_{n'2}}(-1)^{\delta_{k'2}\delta_{n2}}}{4}. \tag{28}$$

Therefore, the process matrix elements in Dirac basis can be directly obtained from the sequential weak value $\langle \hat{B}^n \hat{A}^k \rangle^{n'k'}_{\mathrm{w}}$ as

$$\chi^S_{ij} \equiv \chi^S_{nk,n'k'} = \langle \hat{B}^n \hat{A}^k \rangle^{n'k'}_{\mathrm{w}} \times 4p^{n'k'} \times (-1)^{\delta_{k2}\delta_{n'2}}(-1)^{\delta_{k'2}\delta_{n2}}. \tag{29}$$

Note that nk and $n'k'$ are the binary number representations of i and j, respectively.

Constraints on the process matrix in Dirac basis

For the qubit Hilbert space ($d = 2$), the process matrix is a 4×4 matrix and thus it has 32 real elements. However, since the process matrix is Hermitian, it has at most 16 independent real parameters. We can further reduce the number of independent parameters by invoking that the post-selection probability is given by Supplementary Equation (25). The post-selection probability $p^{n'k'}$ depends on the input state $\hat{\rho}_{\text{in}}^{n'}$, the process $\mathcal{E}$, and the final projector $\hat{\Pi}_{\text{s}}^{k'}$. Thus, there are four possible cases for $p^{n'k'}$, and they are explicitly calculated as

$$\begin{aligned}
p^{11} &= (\chi_{11} + \chi_{12}^* + \chi_{13}^* + \chi_{14}^*)/4,\\
p^{12} &= (\chi_{12} + \chi_{22} - \chi_{23}^* - \chi_{24}^*)/4,\\
p^{21} &= (\chi_{13} - \chi_{23} + \chi_{33} - \chi_{34}^*)/4,\\
p^{21} &= (\chi_{14} - \chi_{24} - \chi_{34} + \chi_{44})/4.
\end{aligned} \tag{30}$$

Since $p^{n'k'}$ is probability, it should be a real number. Therefore, we obtain four constraints for imaginary parts of process matrix elements as

$$\begin{aligned}
\text{Im}(\chi_{12}) + \text{Im}(\chi_{13}) + \text{Im}(\chi_{14}) &= 0,\\
\text{Im}(\chi_{12}) + \text{Im}(\chi_{23}) + \text{Im}(\chi_{24}) &= 0,\\
\text{Im}(\chi_{13}) - \text{Im}(\chi_{23}) + \text{Im}(\chi_{34}) &= 0,\\
\text{Im}(\chi_{14}) - \text{Im}(\chi_{24}) - \text{Im}(\chi_{34}) &= 0.
\end{aligned} \tag{31}$$

Note that these constraints must hold regardless of whether the quantum process is a trace preserving map or a trace non-preserving map [4].

Fidelity estimation via d-QPT

The most common method to compare two quantum process is to evaluate the fidelity between two quantum process matrices. The fidelity is defined by

$$\mathcal{F} = \text{Tr}\left[\sqrt{\sqrt{\chi_{\text{ideal}}^S}\chi_{\text{exp}}^S\sqrt{\chi_{\text{ideal}}^S}}\right], \tag{32}$$

where χ_{exp}^S is the experimentally measured quantum process matrix and χ_{ideal}^S is a target ideal operation. The direct quantum process tomography via sequential weak values allows one to estimate the fidelity between a target ideal operation and an actually implemented operation without a standard full quantum process tomography.

In the main text, we have considered two cases, the Hadamard operation and $\mathcal{R}_x$-gate operation which corresponds to a quarter wave plate (QWP) at 45°. The quantum process matrix for the Hadamard operation is given in Dirac basis as

$$\chi_{\text{Hada}}^S = \frac{1}{2}\begin{pmatrix} 1 & 0 & 0 & 1\\ 0 & 0 & 0 & 0\\ 0 & 0 & 0 & 0\\ 1 & 0 & 0 & 1 \end{pmatrix}. \tag{33}$$

As defined by Supplementary Equation (32), it is straightforward to show that the fidelity for Hadamard operation is evaluated as

$$\mathcal{F} = \sqrt{\text{Re}(\chi_{11}^S + \chi_{44}^S)/2 + \text{Re}(\chi_{14}^S)}. \tag{34}$$

Likewise, the ideal $\mathcal{R}_x$-gate operation is given as

$$\chi_{\mathcal{R}_x\text{-gate}}^S = \frac{1}{4}\begin{pmatrix} 1 & i & 1 & -i\\ -i & 1 & -i & -1\\ 1 & i & 1 & -i\\ i & -1 & i & 1 \end{pmatrix}. \tag{35}$$

And, the fidelity is evaluated as

$$\mathcal{F} = \sqrt{\text{Re}(2\chi_{13}^S - 2\chi_{24}^S + 1)/4 + \text{Im}(\chi_{12}^S - \chi_{34}^S)}, \tag{36}$$

where we have used the constraints of Supplementary Equation (31) and the trace preserving condition.

Compressive sensing quantum process tomography with sequential weak values

The standard full quantum process tomography (s-QPT) is based on the mathematical linear inversion. Therefore, the number of experimental configurations must be larger than the number of independent parameters in the process matrix. In other words, the experimental configurations should be tomographically complete. However, when the dimension of system gets larger, the s-QPT becomes unfavorable as the number of configurations scales exponentially. To resolve this problem, a mathematical technique, known as compressive sensing, has been employed to the quantum process tomography [5, 6].

In the compressive sensing quantum process tomography (cs-QPT), experimentally measured outcomes $\vec{P} = \{P_1, P_2, \cdots, P_n\}$ with n different configurations are used to reconstruct a process matrix, where d is the dimension of the system and thus the process matrix has $m \equiv d^4$ independent parameters. Let us represent the process matrix elements in a vectorized form $\vec{\chi}$, which has at least m elements. $\vec{P}$ and $\vec{\chi}$ have a relation as

$$\vec{P}_{\{n\}} = \Lambda_{\{n\times m\}}\vec{\chi}_{\{m\}}, \tag{37}$$

where the subscripts denote the dimension and Λ is a $n\times m$ matrix given by the experimental configurations. Therefore, if $n < m$, the experimental configurations are tomographically incomplete, thus $\vec{\chi}$ becomes underdetermined. However, the compressive sensing technique allows one to find $\vec{\chi}$ with incomplete set of measurements, where $\vec{\chi}$ is assumed to be sparse. In quantum information processing, it is typically aimed to implement a quantum gate, which is a unitary process and thus the sparsity assumption is valid. The idea of compressive sensing is to solve the following convex optimization problem [5, 6],

$$\text{minimize } ||\vec{\chi}||_1, \tag{38}$$

$$\text{subject to } ||\vec{P} - \Lambda\vec{\chi}||_2 < \epsilon \tag{39}$$

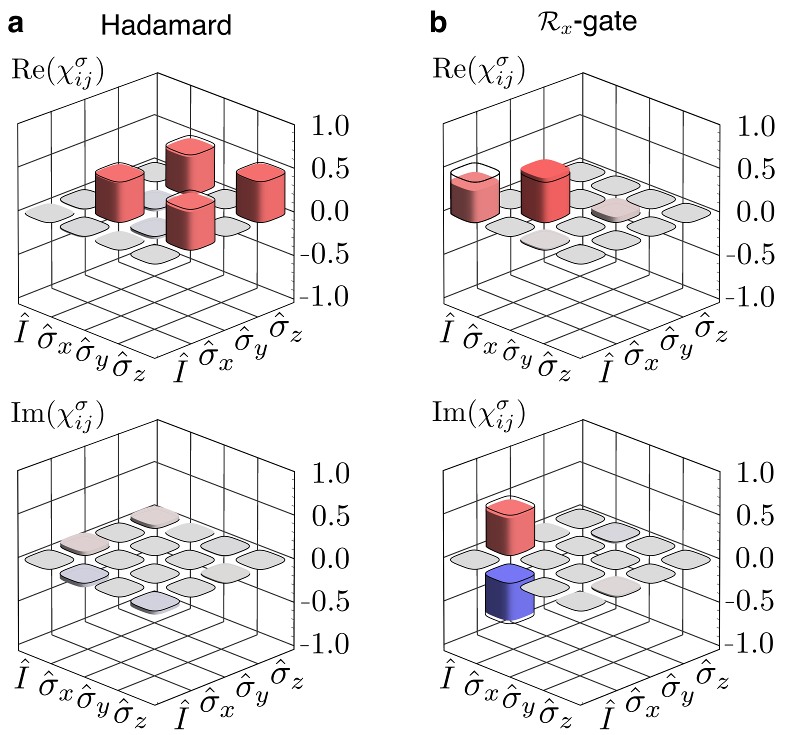

Supplementary Figure 2. **Compressive sensing quantum process tomography with sequential weak values. a** Hadamard operation by HWP at 22.5°. **b** $\mathcal{R}_x$-gate operation by QWP at 45°. Tomographically incomplete set of measurements (only four) is used to reconstruct the process matrix in Pauli basis via the compressive sensing quantum process tomography. Color filled (transparent) bars represent process matrix obtained via the cs-QPT (ideal process matrix). The fidelities are **a** $\mathcal{F} = 0.962 \pm 0.033$ and **b** $\mathcal{F} = 0.917 \pm 0.058$. The errors in $\mathcal{F}$ are obtained by performing 500 Monte-Carlo simulation runs with taking into account the statistical errors in measured weak values.

and the trace preserving condition, where ϵ is the noise bound, $||\cdot||_1$ is the L_1 norm, and $||\cdot||_2$ is the Euclidean L_2 norm.

We employ the compressive sensing QPT technique with our d-QPT results, where elements of $\vec{P}$ are arbitrary chosen by some of the process matrix elements in Dirac basis. It is important to take the basis for the process matrix so that $\vec{\chi}$ is maximally sparse. In experiments, we considered single-qubit rotating operations, such as Hadamard operation and $\mathcal{R}_x$-gate operation. Since the eigen-bases of such rotating operations are Pauli operators, the process matrices of the rotating operations are maximally sparse in Pauli basis. Therefore, we take $\vec{\chi}$ to be the process matrix elements in Pauli basis. And, Λ is set from the transformation relation between the process matrices in Dirac basis and in Pauli basis. Supplementary Figure **2** shows the results of cs-QPT with only four measurement outcomes, where $\vec{P}$ is arbitrary chosen as $\vec{P} = [\mathrm{Re}(\chi^S_{14}), \mathrm{Re}(\chi^S_{33}), \mathrm{Im}(\chi^S_{12}), \mathrm{Im}(\chi^S_{14})]^T$. As shown in Supplementary Figure **2**, cs-QPT gives very convincing results with fidelities of $\mathcal{F} = 0.962 \pm 0.033$ for Hadamard operation and of $\mathcal{F} = 0.917 \pm 0.058$ for $\mathcal{R}_x$-gate operation.

SUPPLEMENTARY REFERENCES

[1] Brodutch, A. & Cohen, E. Nonlocal Measurements via Quantum Erasure. *Phys. Rev. Lett.* **116,** 070404 (2016).

[2] Salvail, J. Z. *et al.* Full characterization of polarization states of light via direct measurement. *Nat. Photonics* **7,** 316–321 (2013).

[3] Hofmann, H. F. Complex joint probabilities as expressions of reversible transformations in quantum mechanics. *New J. Phys.* **14,** 043031 (2012).

[4] Bhandari, R & Peters, N. A. On the general constraints in single qubit quantum process tomography. *Sci. Reports* **6,** 26004 (2016).

[5] Shabani, A. *et al.* Efficient Measurement of Quantum Dynamics via Compressive Sensing. *Phys. Rev. Lett.* **106,** 100401 (2011).

[6] Riofrío, C. A. *et al.* Experimental quantum compressed sensing for a seven-qubit system. *Nat. Commun.* **8,** 15305 (2017).
